# A Hyaluronic Acid-Coated Ethosomal Delivery System for Improving the Topical Delivery of Glycyrrhetinic Acid in Sensitive Skin

**DOI:** 10.3390/pharmaceutics18091114

**Published:** 2026-09-04

**Authors:** Yuling Wang, Shujing Ren, Jun Deng, Dan Luo, Rui Liu, Yu Zhou, Siyuan Chen, Wei Liu

**Affiliations:** 1 College of Life Science and Technology, Huazhong University of Science and Technology, Wuhan 430074, China; wangyl@bloomagebiotech.com (Y.W.);; 2 Research and Development Center, Bloomage Biotechnology Corporation Limited, Jinan 250101, China; 3 National Engineering Research Center for Nanomedicine, Huazhong University of Science and Technology, Wuhan 430075, China; 4 Research Institute for Biomaterials, Tech Institute for Advanced Materials Bioinspired Biomedical Materials & Devices Center, College of Materials Science and Engineering, Jiangsu Collaborative Innovation Center for Advanced Inorganic Function Composites, Suqian Advanced Materials Industry Technology Innovation Center, Nanjing Tech University, Nanjing 211816, China

**Keywords:** sensitive skin, glycyrrhetinic acid, hyaluronic acid, ethosomes, skin delivery

## Abstract

**Background:** Effective topical management of sensitive skin remains challenging because inadequate cutaneous delivery limits the therapeutic performance of many anti-inflammatory agents. Glycyrrhetinic acid (GA) possesses well-recognized anti-inflammatory and barrier-protective activities, yet its clinical potential is constrained by poor aqueous solubility and inefficient skin delivery. This study aimed to develop a hyaluronic acid (HA)-engineered ethosomal system to enhance the local delivery and therapeutic efficacy of GA for sensitive skin. **Methods:** HA-coated GA-loaded ethosomes (HAGA-ETs) were prepared by electrostatic adsorption of HA onto a cationic ethosomal template. The physicochemical properties, release behavior, storage stability, skin retention, cellular uptake, and biological activities of HAGA-ETs were systematically evaluated using TNF-α/IFN-γ-stimulated HaCaT cells and an SLS-induced 3D reconstructed skin model. **Results:** HAGA-ETs exhibited a mean particle size of 140.1 nm, encapsulation efficiency exceeding 95%, sustained drug release, and good storage stability. Compared with Free-GA and unmodified ethosomes, HAGA-ETs showed improved cytocompatibility, enhanced skin retention, greater keratinocyte uptake, and stronger anti-inflammatory activity. HA pre-saturation attenuated the enhanced cellular uptake of HAGA-ETs, supporting the involvement of HA receptor-mediated cellular interaction. HAGA-ETs also more effectively restored barrier-related markers, suppressed hyper-reactivity- and allergy-associated mediators, and inhibited the activation of MAPK/NF-κB, JAK1/STAT1, and TRPV1-related signaling pathways in both cellular and 3D skin models. **Conclusions:** HA surface engineering effectively improved the topical delivery and local therapeutic efficacy of GA by enhancing skin retention and keratinocyte interaction. HAGA-ETs represent a promising nanoplatform for the local management of sensitive skin.

## 1. Introduction

Sensitive skin (SS) is a highly prevalent condition characterized by unpleasant sensations such as burning, stinging, itching, tightness, and dryness, sometimes accompanied by visible signs including erythema and scaling [1,2]. These symptoms may be triggered by otherwise innocuous stimuli, including cosmetics, temperature fluctuations, ultraviolet radiation, and air pollutants [3,4]. Although sensitive skin is not classified as an independent dermatological disease, it is increasingly recognized as an important clinical and cosmetic concern because of its high prevalence and substantial impact on quality of life [5,6].

The pathophysiology of sensitive skin is complex and has not yet been fully elucidated. Current evidence suggests that it involves three closely interconnected processes: skin barrier dysfunction, inflammatory and immune activation, and neural hyper-reactivity [7,8,9]. Among these, barrier impairment is widely considered a central event [10]. The stratum corneum and intercellular lipids together constitute the primary defensive barrier of the skin, maintaining epidermal hydration and limiting the penetration of irritants and allergens [11]. Once this barrier is disrupted, transepidermal water loss increases and the entry of external stimuli is facilitated, which in turn activates keratinocytes and immune cells and promotes the release of pro-inflammatory mediators such as tumor necrosis factor-α (TNF-α), interleukin (IL)-1β, IL-6, and IL-8 [12,13]. In addition, transient receptor potential (TRP) channels, particularly TRPV1 and TRPV4, are closely involved in the perception of burning, stinging, and itching [14,15,16]. Their abnormal activation lowers the sensory threshold of the skin and amplifies responses to weak external stimuli. Meanwhile, Th2-associated mediators such as IL-4, IL-31, and thymic stromal lymphopoietin (TSLP) may further aggravate inflammation and sensory discomfort through neuroimmune interactions [17,18,19]. Therefore, sensitive skin is now generally regarded as a multifactorial condition arising from the interplay of barrier damage, inflammation, and neural sensitization, rather than from a single isolated defect.

Because these pathological processes are mechanistically intertwined, successful topical management of sensitive skin requires therapeutic agents capable of regulating multiple pathological events while maintaining sufficient local exposure within the skin. Glycyrrhetinic acid (GA), a pentacyclic triterpenoid derived from licorice, has been widely reported to possess anti-inflammatory, anti-allergic, and antioxidant activities [20,21]. Previous studies have shown that GA can modulate multiple signaling pathways involved in skin inflammation, including NF-κB and MAPK, thereby reducing the production of inflammatory mediators. These properties make GA a promising candidate for the topical management of sensitive skin. However, GA has poor water solubility and limited skin permeability, and its therapeutic potential in sensitive skin is therefore constrained primarily by inadequate cutaneous delivery rather than by insufficient pharmacological activity [22]. An efficient topical delivery system is therefore essential to fully exploit the therapeutic potential of GA.

Conventional liposomes have been widely used to improve the solubility and skin deposition of poorly water-soluble actives, owing to their phospholipid bilayer structure, which resembles that of skin lipids [23,24,25,26,27]. However, their relatively rigid bilayer limits deformability within the densely packed lipid matrix of the stratum corneum, constraining cutaneous penetration [28]. Ethosomes, which incorporate ethanol into the bilayer, exhibit greater deformability and stronger interaction with stratum corneum lipids, and have accordingly been used to improve the penetration and intradermal deposition of poorly soluble compounds such as GA [29]. Binary ethosomes have subsequently been developed by incorporating propylene glycol together with ethanol, thereby reducing the reliance on high ethanol concentrations while retaining the advantages of alcohol-containing deformable vesicles [30]. Although ethosomes improve cutaneous penetration, strategies capable of simultaneously enhancing local retention, cellular interaction, and biocompatibility remain limited, and penetration alone may not be sufficient to support the localized, sustained action required for sensitive skin. Further engineering of the carrier surface is therefore needed to extend the benefits of ethosomes beyond penetration.

Hyaluronic acid (HA) is a naturally occurring polysaccharide widely distributed in the skin and extracellular matrix. It possesses excellent biocompatibility, strong water-binding capacity, and beneficial effects on skin hydration and barrier recovery [31]. More importantly, HA can interact with HA-binding receptors, including CD44, expressed on skin cells, which may contribute to the adhesion, retention, and cellular interaction of HA-functionalized nanocarriers in cutaneous tissue [32,33]. These characteristics make HA an attractive surface-engineering material for extending the benefits of ethosomes beyond penetration, by improving skin compatibility and local cellular interaction [34]. Despite growing interest in HA-functionalized nanocarriers for topical delivery, few studies have specifically explored how HA-mediated surface engineering can be rationally integrated with ethosomes for sensitive skin, where efficient retention, cellular interaction, and biocompatibility are simultaneously required.

Herein, HA-coated GA-loaded ethosomes (HAGA-ETs) were developed using an electrostatic surface-engineering strategy with the expectation that HA-mediated surface modification would improve local retention, facilitate HA receptor-mediated keratinocyte interaction, and thereby improve the therapeutic efficacy of GA against inflammation, barrier dysfunction, and neurosensory hyper-reactivity in sensitive skin. A cationic ethosome was first prepared to enable electrostatic adsorption of HA onto the vesicle surface, followed by surface charge reversal after HA deposition to yield an HA-coated ethosomal carrier. Compared with covalent conjugation, this electrostatic adsorption strategy preserves the native structure of HA and avoids multistep chemical reactions as well as potential concerns associated with coupling reagents or solvent residues, providing a facile and scalable surface-engineering approach for topical nanocarriers [35]. Building on our previous work, in which competitive HA pre-saturation was used to investigate HA-associated cellular uptake of HA-decorated cationic nanocarriers in keratinocytes [32], the present study extended this surface-engineering strategy to an HA-coated ethosomal system. The physicochemical properties, in vitro release behavior, skin retention, and cellular uptake of the formulation were systematically investigated. Its effects on inflammatory responses, barrier-related proteins, and hyper-reactivity-associated markers were further evaluated in TNF-α/IFN-γ-stimulated HaCaT cells and an SLS-induced 3D skin model. This study establishes a biocompatible, surface-engineered nanoplatform for enhancing the local therapeutic efficacy of GA in sensitive skin.

## 2. Materials and Methods

### 2.1. Materials

Glycyrrhetinic acid was obtained from Fanzhi Pharmaceutical Co., Ltd. (Gansu, China). Hydrogenated lecithin was purchased from ReloPharm Co., Ltd. (Seoul, Korea). Cholesterol and ethanol were supplied by Sinopharm Chemical Reagent Co., Ltd. (Shanghai, China). Stearamidopropyl dimethylamine was purchased from BASF SE (Ludwigshafen am Rhein, Germany). Octyldodecanol was obtained from ERCA (Cuneo, Italy). Propylene glycol was obtained from Dow Chemical Co., Ltd. (Midland, TX, USA). Hyaluronic acid was purchased from Bloomage Biotechnology (Shandong, China). Tween 80, sodium lauryl sulfate (SLS), Dulbecco’s modified Eagle’s medium (DMEM), penicillin–streptomycin solution, and fetal bovine serum (FBS) were obtained from Procell Life Science & Technology Co., Ltd. (Wuhan, China). TNF-α, IFN-γ, and rhodamine B (RhoB) were purchased from MedChemExpress LLC (Monmouth Junction, NJ, USA). ELISA kits for TNF-α, IL-1β, IL-6, IL-8, aquaporin-3 (AQP3), loricrin (LOR), filaggrin (FLG), TRPV1, TSLP, TRPV4, IL-4, and IL-31 were obtained from Meimian Industrial Co., Ltd. (Yancheng, China). Primary and secondary antibodies against NF-κB, p-JAK1, p-MAPK, p-NF-κB, p-STAT1, and p-TRPV1 were purchased from Servicebio Technology Co., Ltd. (Wuhan, China). Physiological saline (0.9% *w*/*v* NaCl) was obtained from Servicebio Biotechnology Co., Ltd. (Wuhan, China). Fertilized chicken eggs were purchased from Nanjing Zhushun Biotechnology Co., Ltd. (Nanjing, China). EpiKutis 3D skin models were purchased from Guangdong Boxi Biotechnology Co., Ltd. (Dongguan, China). Bama pig skin was purchased from Yourong Biotechnology (Yantai, China), and immortalized human keratinocytes were obtained from the Kunming Cell Bank, Chinese Academy of Sciences (Kunming, China).

### 2.2. Preparation of GA-ETs and HAGA-ETs

HAGA-ETs were designed as a propylene glycol-rich binary ethosomal formulation containing ethanol and propylene glycol as the binary alcohol phase. Unlike classical ethosomes, which typically contain a relatively high concentration of ethanol, binary ethosomes incorporate propylene glycol together with ethanol to modulate vesicle properties and skin delivery [30]. In the present formulation, 8% ethanol and 40% propylene glycol were used as the binary alcohol components. HAGA-ETs were prepared using a phase-mixing method combined with high-pressure homogenization. The formulation parameters were determined based on our preliminary formulation development. SADM and HA concentrations were investigated over the ranges of 0.05–0.30% and 0.1–2.0% (*w*/*w*), respectively, and 0.16% SADM and 1% HA were selected for the final formulation based on the physicochemical properties and coating performance of the resulting ethosomes. In addition, the phospholipid/cholesterol ratio was investigated over the range of 1:1 to 5:1, and 0.2% hydrogenated lecithin and 0.2% cholesterol (1:1, *w*/*w*) were selected based on favorable GA encapsulation efficiency, particle size, and PDI. Briefly, Phase A consisted of hydrogenated lecithin (0.2%, *w*/*w*), cholesterol (0.2%, *w*/*w*), ethanol (8.0%, *w*/*w*) and stearamidopropyl dimethylamine (0.16%, *w*/*w*) dissolved in water under stirring at 70 °C. Phase B consisted of GA (1%, *w*/*w*) and octyldodecanol (10%, *w*/*w*) dissolved at 70 °C. A GA feeding concentration of 1% (*w*/*w*) was selected based on preliminary formulation development, which provided satisfactory drug incorporation and formulation characteristics under the conditions investigated. Phase C was prepared by dissolving propylene glycol (40%, *w*/*w*) in water at 70 °C under continuous stirring. Phase D consisted of HA (1%, *w*/*w*) (MW: 5 kDa) dissolved in water (28.64%, *w*/*w*) under stirring. Phases A and B were mixed thoroughly, followed by the addition of Phase C under constant stirring at 70 °C to obtain a homogeneous mixture at 300 rpm. The resulting dispersion was then processed by high-pressure homogenization (JN-02HC) at 1000–1200 bar for 2–5 cycles at 70 °C. Subsequently, Phase D was added to the homogenized mixture under stirring to yield the final HAGA-ETs formulation. GA-loaded cationic ethosomes were thus formed prior to HA addition, and HA was then adsorbed onto the positively charged vesicle surface through electrostatic interactions. GA-ETs were prepared using the same procedure without the addition of Phase D (HA solution).

### 2.3. Physicochemical Characterization

An appropriate amount of each sample (HAGA-ETs and GA-ETs) was diluted 200-fold with ultrapure water to a suitable concentration, and the average scattering intensity was adjusted to 150–300. The particle size, polydispersity index (PDI), and zeta potential were measured by dynamic light scattering (DLS) using a Zetasizer Nano ZS90 instrument (Malvern Instruments, Malvern, UK). Measurements were performed at 25 °C with a scattering angle of 90°. The morphology of GA-ETs and HAGA-ETs was examined by transmission electron microscopy (TEM; HT7700, Hitachi, Tokyo, Japan). Samples were diluted 200-fold with ultrapure water, placed onto formvar-coated copper grids for 1–2 min, and negatively stained with 2% phosphotungstic acid for 1–2 min. After air-drying at room temperature, the samples were observed under TEM.

Drug loading (DL) and encapsulation efficiency (EE) were determined by ultrafiltration centrifugation. Briefly, 0.5 mL of each ethosomal suspension was transferred into an ultrafiltration tube (molecular weight cutoff: 3 kDa) and centrifuged at 12,000 rpm for 30 min to separate Free-GA from the vesicle-associated fraction. The filtrate was collected, and the amount of Free-GA was quantified by HPLC. For determination of the total GA content, another 0.5 mL of the same sample was mixed with methanol to disrupt the vesicles and then brought to a final volume of 4 mL, followed by HPLC analysis. GA was quantified using a Sepax Bio-C18 column on an HPLC system (BOCL 101, SHIMADZU, Kyoto, Japan). The mobile phase consisted of methanol and 0.1% phosphoric acid aqueous solution (80:20, *v*/*v*) at a flow rate of 1.0 mL/min. The column temperature was maintained at 35 °C, the detection wavelength was set at 250 nm, and the injection volume was 10 μL according to a previously reported method [36]. The analytical method was validated in terms of linearity, instrumental precision, sample stability, and repeatability. A good linear relationship was obtained for GA over the concentration range of 0.488–125 μg/mL, with R^2^ of 0.9997. The RSD of the peak area for instrumental precision was 0.261%. The RSDs for sample stability over 48 h were 0.642% and 0.572% for GA-ETs and HAGA-ETs, respectively. The repeatability RSDs were 0.409% and 0.314%, respectively. DL and EE were calculated using the following equations [37]:

(1) $$\mathrm{DL}(\%)\;=\;\frac{\mathrm{We}}{\mathrm{Wm}}\times \;100\%\;$$

(2) $$\mathrm{EE}(\%)=\frac{\mathrm{We}}{\mathrm{We}+\mathrm{Wf}}\times \;100\%$$

where We is the mass of GA encapsulated in ethosomes, Wm is the total mass of the ethosomal formulation, and Wf is the mass of Free-GA.

### 2.4. HET-CAM Assay for Irritation Assessment

The Hen’s egg test—chorioallantoic membrane (HET-CAM) assay was performed according to the OECD Test Guideline 438 using fertilized chicken eggs at embryonic day 9. After candling to identify the air chamber, the eggshell was disinfected and a small window was opened at the air chamber end to expose the chorioallantoic membrane (CAM). HAGA-ETs and GA-ETs stock solutions were diluted 10-fold with physiological saline, and 0.2 mL of each diluted formulation was applied directly to the CAM surface. Physiological saline and 0.1% NaOH were used as the negative and positive controls, respectively. The CAM was continuously observed for 5 min, and the onset times of hyperemia, hemorrhage, and coagulation were recorded. Six eggs were used for each group. The irritation score (IS) was calculated based on the recorded response times, and the samples were classified according to the criteria specified in OECD Test Guideline 438 [38].

(3) $$\mathrm{IS}=\frac{\left[(301-\sec H)\times 5+(301-\sec L)\times 7+(301-\sec C)\times 9\right]}{300}$$

where *secH* is the time to onset of hyperemia (s), *secL* is the time to onset of hemorrhage (s), and *secC* is the time to onset of coagulation (s). The irritation scores were classified as follows: 0–0.9, no irritation; 1.0–4.9, slight irritation; 5.0–8.9, moderate irritation; 9.0–21.0, severe irritation.

### 2.5. In Vitro Release Study

The in vitro release of GA from different formulations was evaluated using a dialysis bag method. Equal volumes (1 mL) of Free-GA, GA-ETs, and HAGA-ETs containing equivalent amounts of GA were separately loaded into dialysis bags (molecular weight cutoff: 3 kDa), air bubbles were removed, and the bags were tightly sealed. Each dialysis bag was immersed in 80 mL of PBS (pH 7.4) containing 1% Tween 80 and incubated at 37 °C with shaking at 120 rpm. At 2, 4, 6, 8, 10, 12, 24, and 48 h, 1 mL of release medium was withdrawn and immediately replaced with an equal volume of fresh prewarmed medium. The concentration of GA in the collected samples was determined by HPLC, and the cumulative release percentage was calculated accordingly [39].

(4) $$Q(\%)=\frac{CnV+\sum_{i=1}^{n-1}CiVs}{M0}\times 100\%$$

where Q is cumulative release percentage, *Cn* is drug concentration at the nth sampling time point, *Ci* is drug concentration at the ith sampling time point (i ≤ *n* − 1), *V* is the total volume of the release medium, *Vs* is volume of each withdrawn sample, and *M*0 is the total amount of drug in the dialysis bag.

### 2.6. Ex Vivo Skin Permeation and Retention Study

Ex vivo skin retention of GA was evaluated using Franz diffusion cells. Bama miniature pig skin, purchased from Yourong Biotechnology (Yantai, China), was excised from the dorsal region and mounted between the donor and receptor chambers with the stratum corneum facing the donor compartment. Free-GA, GA-ETs, and HAGA-ETs (0.5 g each; equivalent GA dose) were applied uniformly to the skin surface. The receptor chamber was filled with 7 mL PBS (pH 7.4) containing 1% Tween 80 and maintained at 32 °C under continuous stirring at 300 rpm. At 1, 2, 4, 6, 8, 10, 12, and 24 h, 1 mL of receptor medium was withdrawn and immediately replaced with an equal volume of fresh prewarmed medium. The collected receptor samples were analyzed by HPLC to determine the amount of GA that had permeated through the skin. No detectable GA was observed in the receptor phase for any formulation at any sampling time point, with an LOD of 0.2 μg/mL. After 24 h, the skin was removed, gently rinsed three times with ultrapure water to eliminate residual sample on the surface, and blotted dry with filter paper. The skin was then cut into pieces and homogenized thoroughly with 2 mL of methanol using a tissue homogenizer. The homogenate was transferred to a centrifuge tube and centrifuged at 12,000 rpm for 20 min. The supernatant was filtered through a 0.45 μm membrane and analyzed by HPLC to determine the amount of GA retained in the skin. The skin retention per unit area was calculated as follows:

(5) $$Skin\;retention=\frac{C\times V}{S}$$

where C is the measured GA concentration in the extract, V is the total volume of the extraction solvent, and S is the effective permeation area (2.27 cm^2^).

For visualization of skin distribution, RhoB was used as a fluorescent probe instead of GA, and fluorescent ethosomes were prepared using the same procedure. Free-RhoB, RhoB-ETs, and HARhoB-ETs (0.5 g each; RhoB concentration 100 mg/L) were applied to the skin using the same Franz diffusion setup. At 2 and 4 h, the skin was removed, and residual sample on the surface was gently wiped off with a cotton swab. The skin was then rinsed three times with PBS and blotted dry with filter paper. After embedding, the skin was vertically cryosectioned into slices with a thickness of 8–9 μm. The fluorescence distribution in different skin layers was observed and photographed under a fluorescence microscope (IX71, Olympus, Tokyo, Japan). The fluorescence intensity was semi-quantitatively analyzed using ImageJ software (Image J win-java8), and the mean fluorescence intensity was calculated from at least three different fields for each group [39].

### 2.7. Cell Culture

HaCaT cells were cultured in DMEM supplemented with 10% FBS and 1% penicillin–streptomycin at 37 °C in a humidified atmosphere containing 5% CO_2_. Cells in the logarithmic growth phase were used for subsequent experiments.

### 2.8. In Vitro Cytotoxicity and Cell Viability Under Inflammatory Conditions

HaCaT cells were seeded into 96-well plates at a density of 1 × 10^5^ cells/mL (100 μL/well) and incubated overnight. For cytotoxicity evaluation, the cells were treated at concentrations of 15.63, 31.25, 62.50, 125.00, 250.00, 500.00, and 1000.00 μg/mL for 24 h, with the GA content fixed at 1% in all test formulations, while untreated cells served as the normal control. To assess cell viability under inflammatory conditions, the cells were co-incubated with TNF-α and IFN-γ (10 ng/mL each) together with Free-GA, GA-ETs, or HAGA-ETs at 15.63 μg/mL for 24 h. After treatment, the cells were washed with PBS and incubated with 10% (*v*/*v*) CCK-8 solution for 2 h, and cell viability was measured according to the manufacturer’s instructions [40].

### 2.9. Cellular Uptake Study

For qualitative analysis, HaCaT cells were seeded in 35 mm glass-bottom dishes at a density of 3 × 10^5^ cells/dish and incubated overnight. Cells were then treated with Free-RhoB, RhoB-ETs, or HARhoB-ETs at an equivalent RhoB concentration of 2 μg/mL for 2 or 4 h. After incubation, cells were washed three times with cold PBS, fixed with 4% paraformaldehyde for 15 min, stained with DAPI (2 μg/mL) for 15 min in the dark, and observed using a confocal laser scanning microscope (CLSM; FV3000, Olympus, Tokyo, Japan). For quantitative analysis, HaCaT cells were seeded into 6-well plates at a density of 3 × 10^5^ cells/well and treated under the same conditions. After incubation, cells were washed with PBS, trypsinized, centrifuged, and resuspended in 0.5 mL PBS before analysis by flow cytometry (CytoFLEX, Beckman Coulter, Brea, CA, USA). Cellular uptake was expressed as mean fluorescence intensity [41].

### 2.10. Competitive HA Pre-Saturation Study

HaCaT cells in the logarithmic growth phase were seeded into 12-well plates at a density of 2 × 10^5^ cells/well and cultured for 24 h. To investigate whether HA-mediated cell-surface recognition contributed to the uptake of HA-coated ethosomes, one group of cells was preincubated with excess HA (360 kDa, 10 mg/mL) in serum- and antibiotic-free medium for 1 h, whereas the control group received no HA pretreatment. The HA molecular weight and concentration were selected according to previous study [32]. High-molecular-weight HA was used to competitively occupy HA-binding sites on the cell surface. Cells were subsequently incubated with FITC-labeled HA (5 kDa) in serum-free medium at 37 °C for 4 h. The low-molecular-weight FITC-HA served as a fluorescent probe to visualize HA uptake. After incubation, the cells were washed three times with PBS, fixed with 4% paraformaldehyde for 15 min, and observed by CLSM.

To further evaluate the effect of HA pre-saturation on the uptake of HA-coated ethosomes, cells pretreated as described above were incubated with Free-RhoB, RhoB-ETs, or HARhoB-ETs (RhoB concentration: 2 μg/mL) for 4 h. The cells were then washed three times with PBS, fixed with 4% paraformaldehyde for 15 min, and visualized by CLSM.

### 2.11. Cytokine and Protein Quantification in HaCaT Cells

HaCaT cells were seeded into 24-well plates at 1 × 10^5^ cells/well (1 mL/well) and cultured overnight. After removal of the original medium, cells were divided into five groups (n = 5 wells per group): normal control (NC), model control (MC), Free-GA, GA-ETs, and HAGA-ETs. The normal control group received complete DMEM, whereas the model control group was treated with complete DMEM containing TNF-α and IFN-γ (10 ng/mL each). The Free-GA, GA-ETs, and HAGA-ETs groups were treated with complete DMEM containing TNF-α and IFN-γ (10 ng/mL each) together with the corresponding formulations at 15.63 μg/mL. After 24 h, culture supernatants and cell lysates were collected for ELISA-based quantification of pro-inflammatory cytokines (IL-6, IL-8, IL-1β, and TNF-α), allergy-related mediators (IL-31, IL-4, TRPV1, TSLP, and TRPV4), and barrier-associated proteins (FLG, LOR, and AQP3) [42].

### 2.12. Western Blotting

HaCaT cells were seeded into 6-well plates at a density of 2 × 10^5^ cells/mL (2 mL/well) and cultured until 90% confluence before treatment. Cells were then treated as described in Section 2.11. Total protein was extracted using RIPA lysis buffer, and protein concentrations were determined using a BCA protein assay kit. Equal amounts of protein (20–30 μg) were separated by 12% SDS-PAGE and transferred onto PVDF membranes. The membranes were blocked with protein-free blocking solution (G2052) and incubated overnight at 4 °C with primary antibodies against p-NF-κB, NF-κB, p-MAPK, p-JAK1, p-STAT1, and p-TRPV1. After washing with TBST, the membranes were incubated with the corresponding HRP-conjugated secondary antibodies for 1 h at room temperature. Protein bands were visualized using an ECL detection kit and quantified with ImageJ software. β-Actin was used as the loading control. The relative level of p-NF-κB was normalized to total NF-κB, while the relative levels of p-MAPK, p-JAK1, p-STAT1, and p-TRPV1 were normalized to β-actin [42].

### 2.13. Anti-Inflammatory, Anti-Sensitive, and Barrier Repair Efficacy Evaluation in 3D Skin Model

EpiKutis is a three-dimensional reconstructed human skin model that recapitulates key structural and barrier properties of native human skin. In this study, EpiKutis models were used as an in vitro platform to evaluate the anti-inflammatory, anti-irritation, and barrier-repair effects of the formulations. EpiKutis models were removed from the nutrient agar and equilibrated overnight in 6-well plates at 37 °C in a humidified atmosphere containing 5% CO_2_. Three independent experiments were performed to evaluate the anti-inflammatory, anti-sensitivity, and barrier repair effects of the formulations in the 3D skin model. For each experiment, the models were randomly assigned to the normal control (NC), model control (MC), positive control (PC), and treatment groups (Free-GA, GA-ETs, and HAGA-ETs), with three replicates per group. Except for the NC group, all groups were stimulated with 12.5 μL of 0.1% SLS, followed by treatment with the corresponding samples (aqueous formulations containing 1% Free-GA, GA-ETs, or HAGA-ETs) or positive controls for 24 h. For anti-inflammatory evaluation, dexamethasone (100 μg/mL) was used as the positive control, and TNF-α levels in the culture supernatants were determined by ELISA. For anti-sensitivity evaluation, trans-4-tert-butylcyclohexanol (15.6 μg/mL, 12.5 μL) was used as the positive control. For barrier repair evaluation, WY14643 (50.0 μM, 12.5 μL) was used as the positive control. After treatment, the tissues were then fixed with 4% paraformaldehyde for 24 h, paraffin-embedded, sectioned at 6 μm, and subjected to immunofluorescence staining with anti-TRPV1 primary antibody (1:200, Abcam, ab6166) and FITC-conjugated goat anti-rabbit IgG secondary antibody (1:500, Servicebio, GB22303). For barrier repair evaluation, tissue sections were similarly stained with anti-AQP3 primary antibody (1:200, Abcam, ab125219) and Cy3-conjugated goat anti-rabbit IgG secondary antibody (1:500, Servicebio, GB21303). Nuclei were counterstained with DAPI, and fluorescence images were acquired for analysis [43].

### 2.14. Statistical Analysis

All data are presented as mean ± SD from at least three independent experiments. Statistical analyses were performed using one-way analysis of variance (ANOVA) followed by Tukey’s multiple comparisons test in GraphPad Prism 9.5.1. A *p*-value < 0.05 was considered statistically significant.

## 3. Results and Discussion

### 3.1. Physicochemical Characterization of ETs

**Figure 1:**
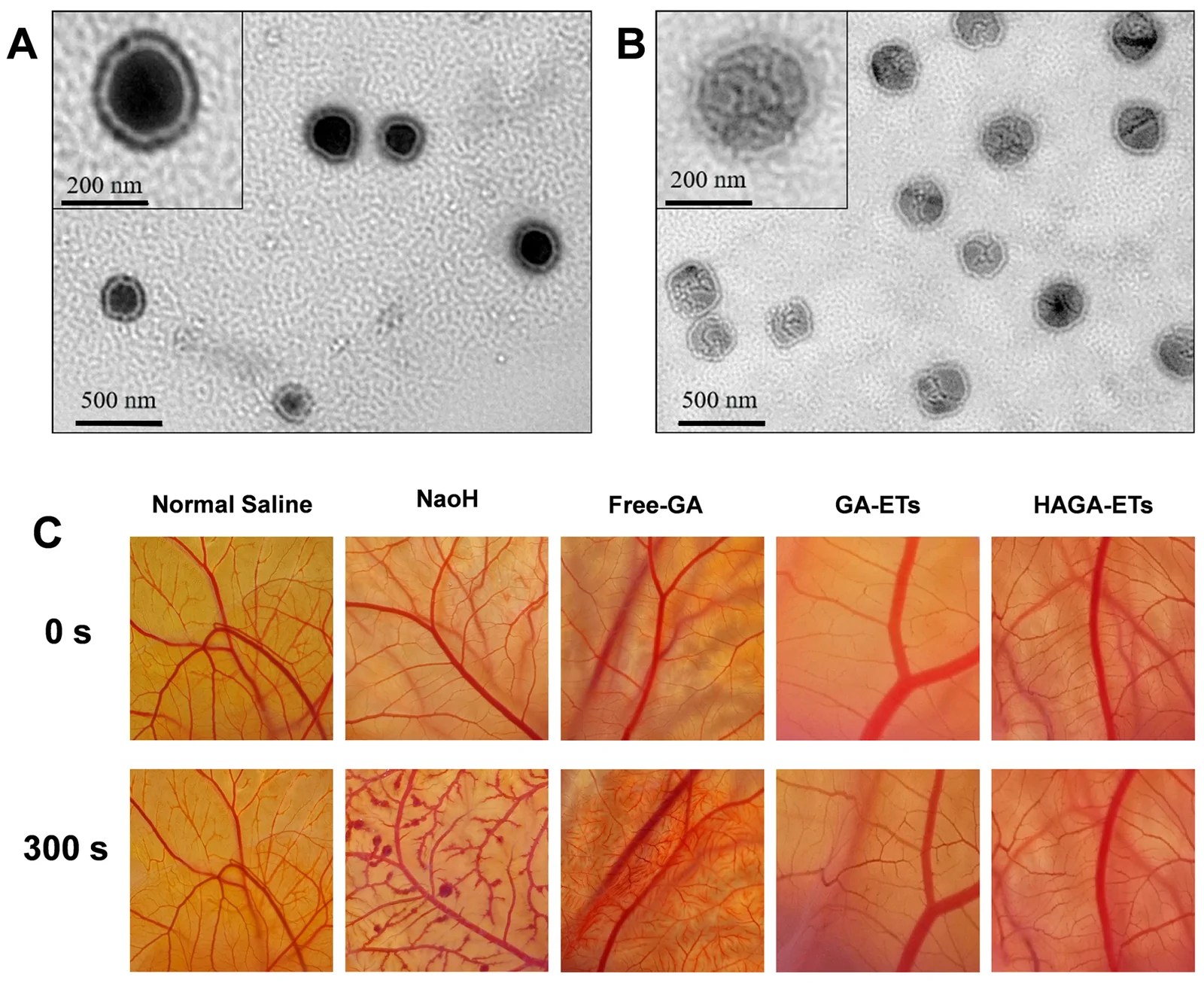
Morphology of GA-ETs and HAGA-ETs. TEM images of (**A**) GA-ETs and (**B**) HAGA-ETs. (**C**) Representative images of the chorioallantoic membrane at 0 and 300 s following treatment with different formulations in the HET-CAM assay.

HAGA-ETs were fabricated by electrostatic adsorption of HA onto preformed GA-loaded cationic ethosomes. As shown in Table 1, compared with GA-ETs, HA coating resulted in a moderate increase in particle size together with complete surface charge reversal from +27.16 ± 0.72 mV to −20.25 ± 0.58 mV, consistent with successful surface coating by HA through electrostatic adsorption. The narrow PDI values (<0.12) suggested good colloidal homogeneity, implying that HA deposition did not induce vesicle aggregation or destabilization (Figure S1). TEM observations confirmed the formation of spherical vesicles with discernible lipid bilayer structures and no obvious aggregation for both formulations (Figure 1). The vesicle dimensions observed by TEM were consistent with the nanoscale size determined by DLS, although the measured values were slightly smaller, as expected owing to dehydration during sample preparation. The mean particle size of approximately 140 nm is well suited for topical application (Figure S1). Nanocarriers within this size range are generally considered favorable for close contact with the stratum corneum and efficient deposition within superficial skin layers, favoring local skin deposition rather than rapid passage across the skin [44]. For sensitive skin, in which barrier dysfunction, inflammatory activation, and neurosensory abnormalities are predominantly confined to the epidermis and upper dermis, such localization is particularly desirable. The physicochemical profile of HAGA-ETs is therefore consistent with their intended role as a localized topical delivery system. The irritation potential of the formulations was further evaluated using the HET-CAM assay according to OECD TG 438 (Figure 1). Free GA exhibited an irritation score of 13.27, corresponding to severe irritation, whereas both GA-ETs and HAGA-ETs showed a markedly lower irritation score of 0.07, which was classified as no irritation. These results indicate that the developed ethosomal formulations exhibited a favorable irritation profile under the experimental conditions, supporting their potential suitability for topical application.

The EE of GA in HAGA-ETs remained above 93%, indicating that electrostatic HA deposition did not compromise vesicle integrity or induce noticeable drug leakage during surface modification. The high EE is likely attributable to the hydrophobic nature of GA, which preferentially partitions into the phospholipid bilayer rather than the aqueous phase. The physicochemical stability of GA-ETs and HAGA-ETs was evaluated during 60 days of storage at 4 °C and 25 °C (Table S1). At 4 °C, both formulations showed relatively limited changes in particle size, PDI, and zeta potential throughout the storage period, indicating good physical stability under refrigerated conditions. In contrast, storage at 25 °C resulted in more pronounced changes, particularly for HAGA-ETs. After 60 days, the mean particle size of HAGA-ETs increased from 140.1 to 177.6 nm, while the PDI increased from 0.115 to 0.246, indicating increased particle-size heterogeneity and some degree of physical destabilization. Therefore, 4 °C is considered more suitable for maintaining the physicochemical properties of HAGA-ETs during storage.

### 3.2. In Vitro Release Behavior

**Figure 2:**
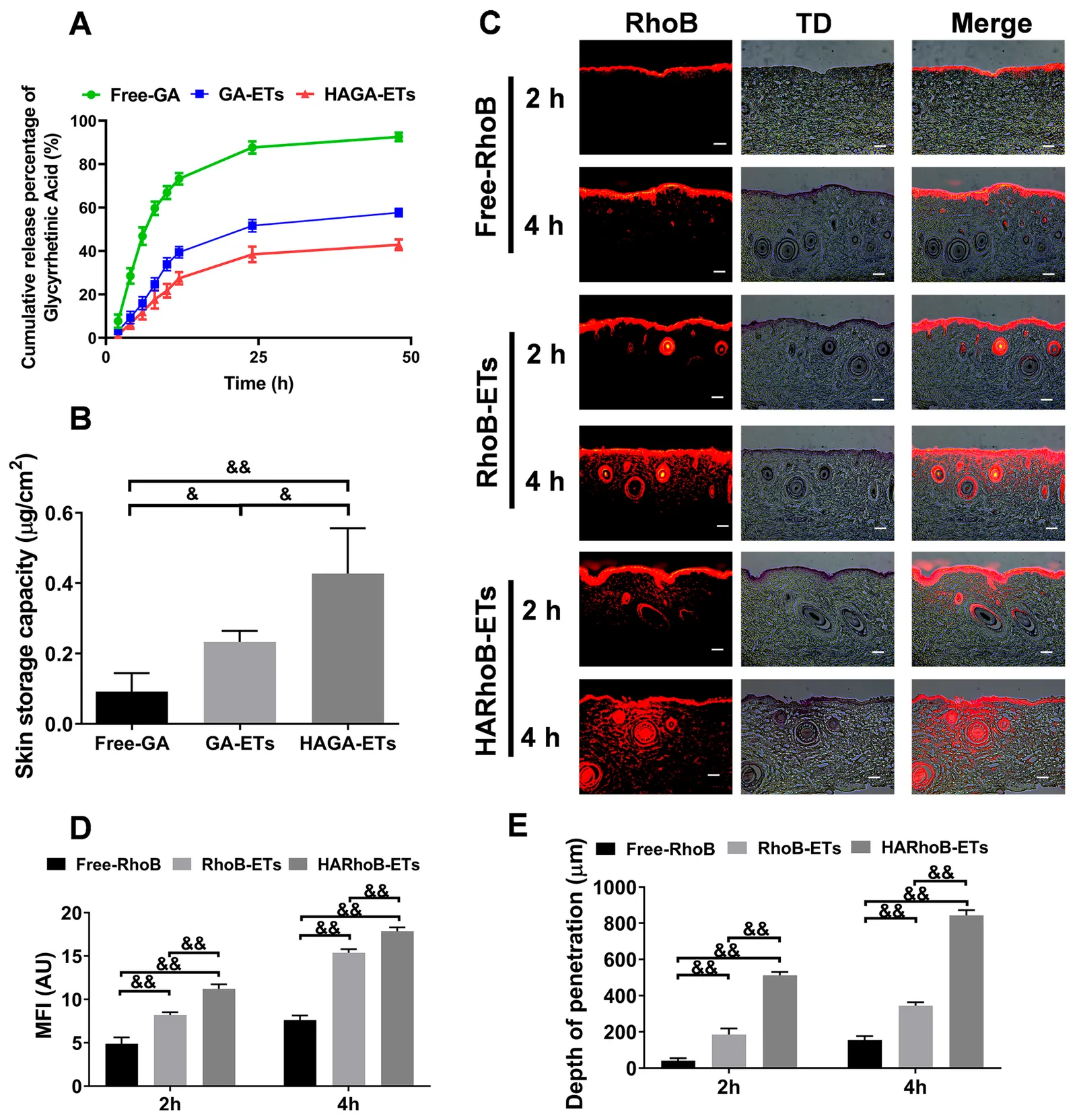
In vitro release, skin retention, and cutaneous distribution of different formulations. (**A**) Cumulative release profiles of Free-GA, GA-ETs, and HAGA-ETs in PBS (pH 7.4) containing 1% Tween 80. (**B**) Skin retention of GA after 24 h in the Franz diffusion cell system. (**C**) Representative fluorescence images showing the skin distribution of free RhoB, RhoB-ETs, and HARhoB-ETs at 2 h and 4 h. (**D**) Quantitative analysis of fluorescence intensity in skin sections. (**E**) Quantitative analysis of penetration depth in skin tissue. Data are presented as mean ± SD (n = 3). ^&^ *p* < 0.05, ^&&^ *p* < 0.01. Scale bar = 200 μm.

The in vitro release profiles of Free-GA, GA-ETs, and HAGA-ETs were evaluated using the dialysis method in PBS (pH 7.4) at 37 °C (Figure 2A). Free-GA exhibited rapid release, reaching a cumulative release of 93.78% within 24 h. Encapsulation into ethosomes markedly slowed drug release, with cumulative release decreasing to 58.46% for GA-ETs, while HAGA-ETs showed the slowest release profile (43.51% at 24 h). These findings indicate that ethosomes encapsulation effectively retarded GA diffusion, and that subsequent HA coating further prolonged drug release. To clarify the fate of the unreleased GA, a mass balance analysis was performed after 48 h. For HAGA-ETs, 43.51%of the initial GA amount was released into the receptor medium, while 55.47% remained associated with the formulation within the dialysis bag, corresponding to a total recovery of 98.98%. In addition, the saturation solubility of GA in PBS containing 1% Tween 80 at 37 °C was determined to be 339.12 μg/mL. The maximum theoretical GA concentration in the receptor medium was approximately 100 μg/mL, corresponding to only 29.5% of the saturation solubility, indicating that the receptor medium remained sufficiently below saturation and that sink conditions were maintained throughout the release experiment.

To further characterize the release kinetics, the experimental data were fitted to four kinetic models, namely zero-order, first-order, Higuchi, and Weibull models, and the comparative fitting results are summarized in Table S2. Among the models tested, the Weibull model consistently yielded the highest coefficients of determination for all three formulations, with R^2^ values of 0.999, 0.998, and 0.999 for Free-GA, GA-ETs, and HAGA-ETs, respectively, exceeding those obtained with the other models. Accordingly, the Weibull model was selected for subsequent quantitative analysis of the release kinetics, and the corresponding fitted parameters are presented in Table 2. The time-scale parameter (τ) progressively increased from Free-GA to GA-ETs and HAGA-ETs, confirming the gradual reduction in release rate following vesicle encapsulation and HA coating. The lower β value of HAGA-ETs further indicated a less pronounced initial release tendency, consistent with the sustained release profile observed experimentally. The sustained release behavior of HAGA-ETs is likely attributable to the combined effects of vesicular encapsulation and HA-mediated diffusion control. Drug molecules must first partition from the lipid bilayer into the aqueous phase before being released into the surrounding medium, whereas the hydrated HA coating may provide an additional diffusional barrier, thereby further prolong drug release. For sensitive skin, sustained rather than rapid drug release is particularly desirable because excessive local exposure may increase irritation risk, whereas controlled release is expected to maintain therapeutic drug levels within the skin while minimizing concentration fluctuations. The sustained release behavior of HAGA-ETs is therefore expected to facilitate prolonged drug retention within the skin, which may contribute to sustained local pharmacological activity while minimizing excessive drug exposure immediately after application.

### 3.3. Ex Vivo Skin Permeation and Retention

No detectable GA was observed in the receptor phase within 24 h for Free-GA, GA-ETs, or HAGA-ETs, indicating negligible transdermal permeation under the experimental conditions. As shown in Figure 2B, both GA-ETs and HAGA-ETs significantly increased GA retention compared with Free-GA, with HAGA-ETs exhibiting the highest retention. The enhanced retention is likely attributable to the well-recognized deformability of ethosomes, which is known to facilitate interaction with stratum corneum lipids, together with the hydration and bioadhesive properties imparted by the HA coating. In addition, HA surface engineering may prolong local residence and strengthen interaction with epidermal cells through HA receptor-mediated recognition. Collectively, these factors contribute to improved localization of GA within the skin. The absence of detectable GA in the receptor phase, together with the substantially enhanced skin retention, indicates that the formulations primarily promoted cutaneous localization rather than transdermal passage. This delivery profile is particularly relevant to the topical management of sensitive skin, for which local drug availability within the epidermis and upper dermis is the intended therapeutic outcome.

RhoB-labeled formulations were further used to visualize the cutaneous distribution of the different formulations. Both RhoB-ETs and HARhoB-ETs exhibited stronger fluorescence signals than free RhoB at both time points (Figure 2C), indicating enhanced skin deposition. HARhoB-ETs consistently showed the highest fluorescence intensity and a more extensive fluorescence distribution within the epidermis and superficial dermis, consistent with the quantitative analysis of fluorescence intensity and distribution depth (Figure 2D,E), further supporting the improved cutaneous localization afforded by HA surface engineering. From a therapeutic perspective, enhanced cutaneous retention is more valuable than unrestricted transdermal permeation for the management of sensitive skin, where pathological changes are predominantly confined to the epidermis and upper dermis. For the topical management of sensitive skin, a formulation that maximizes local drug availability within the epidermis and upper dermis is more rationally aligned with the therapeutic objective than one designed primarily to promote transdermal permeation [45]. Taken together, these findings demonstrate that HA surface engineering optimizes the cutaneous delivery profile of ethosomes by promoting drug localization within disease-relevant skin layers, thereby providing a delivery strategy that is better aligned with the therapeutic requirements of sensitive skin than one focused solely on enhancing transdermal permeation.

### 3.4. In Vitro Cytotoxicity

**Figure 3:**
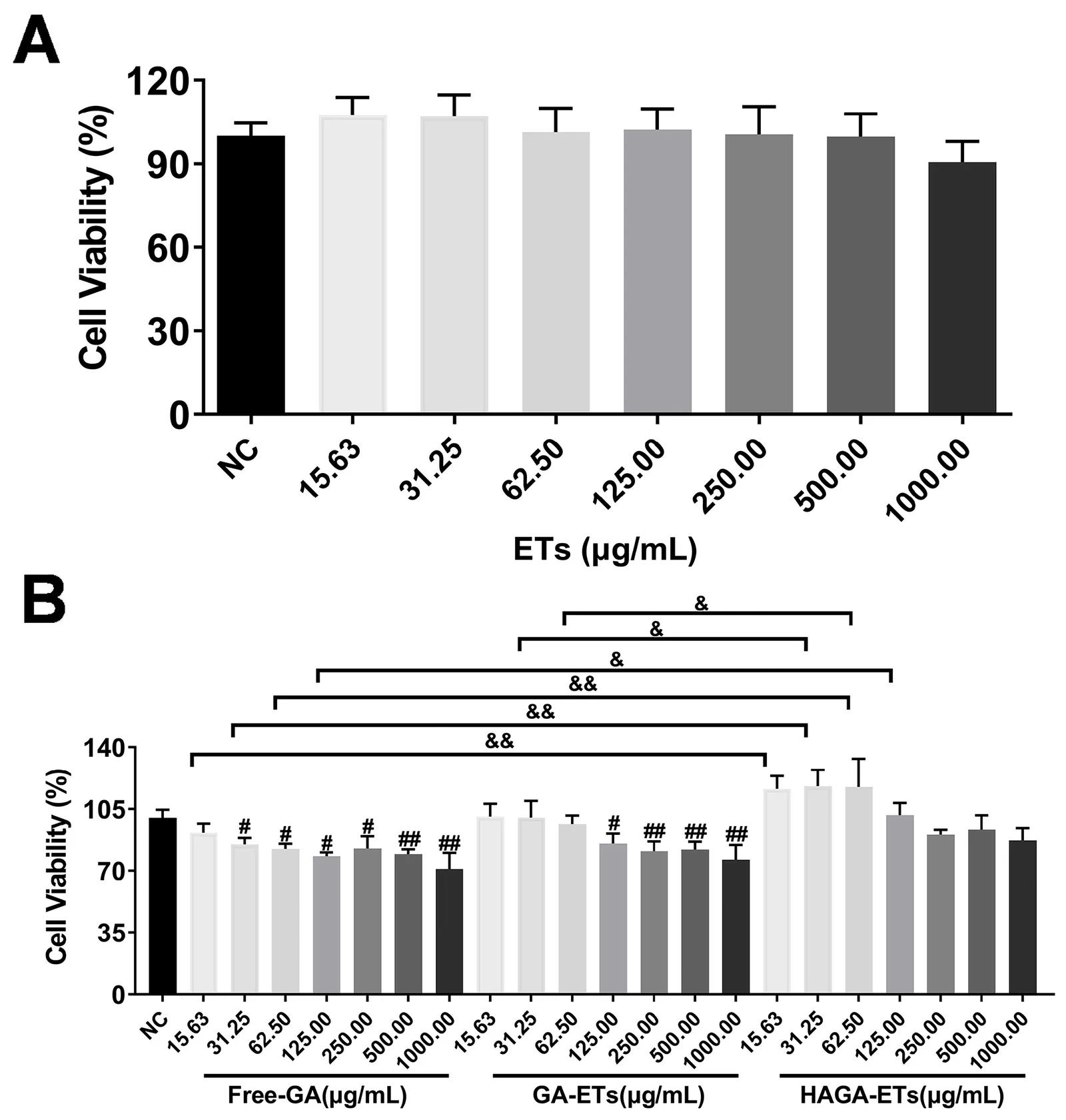
In vitro cytotoxicity of (**A**) ETs, and (**B**) Free-GA, GA-ETs, and HAGA-ETs in HaCaT cells. Data are presented as mean ± SD (n = 5). ^#^ *p* < 0.05, ^##^ *p* < 0.01 vs. NC, ^&^ *p* < 0.05, ^&&^ *p* < 0.01, Mean ± SD (n = 5).

The cytotoxicity of the formulations was assessed in HaCaT cells (Figure 3). ETs without GA loading maintained cell viability above 90% across the entire concentration range tested (Figure 3A), confirming that the ethosomal carrier itself was well tolerated by keratinocytes. Free-GA significantly reduced HaCaT cell viability at 31.25 μg/mL, whereas a significant decrease was observed for GA-ETs only at 125.00 μg/mL and above. In contrast, HAGA-ETs maintained higher cell viability, indicating that HA surface engineering markedly improved biocompatibility. The improved biocompatibility of HAGA-ETs is likely attributable to the shielding effect of the HA coating together with the sustained release behavior of the formulation, both of which reduce direct cellular exposure to GA. For a formulation intended for sensitive skin, carrier biocompatibility is not only a safety requirement but also a prerequisite for reliable pharmacological evaluation. Based on these results, 15.63 μg/mL was selected for subsequent cell experiments, this concentration was chosen to avoid interference from direct cytotoxicity while allowing evaluation of the biological effects of the formulations under inflammatory stimulation.

### 3.5. Cellular Uptake and HA-Mediated Interaction

**Figure 4:**
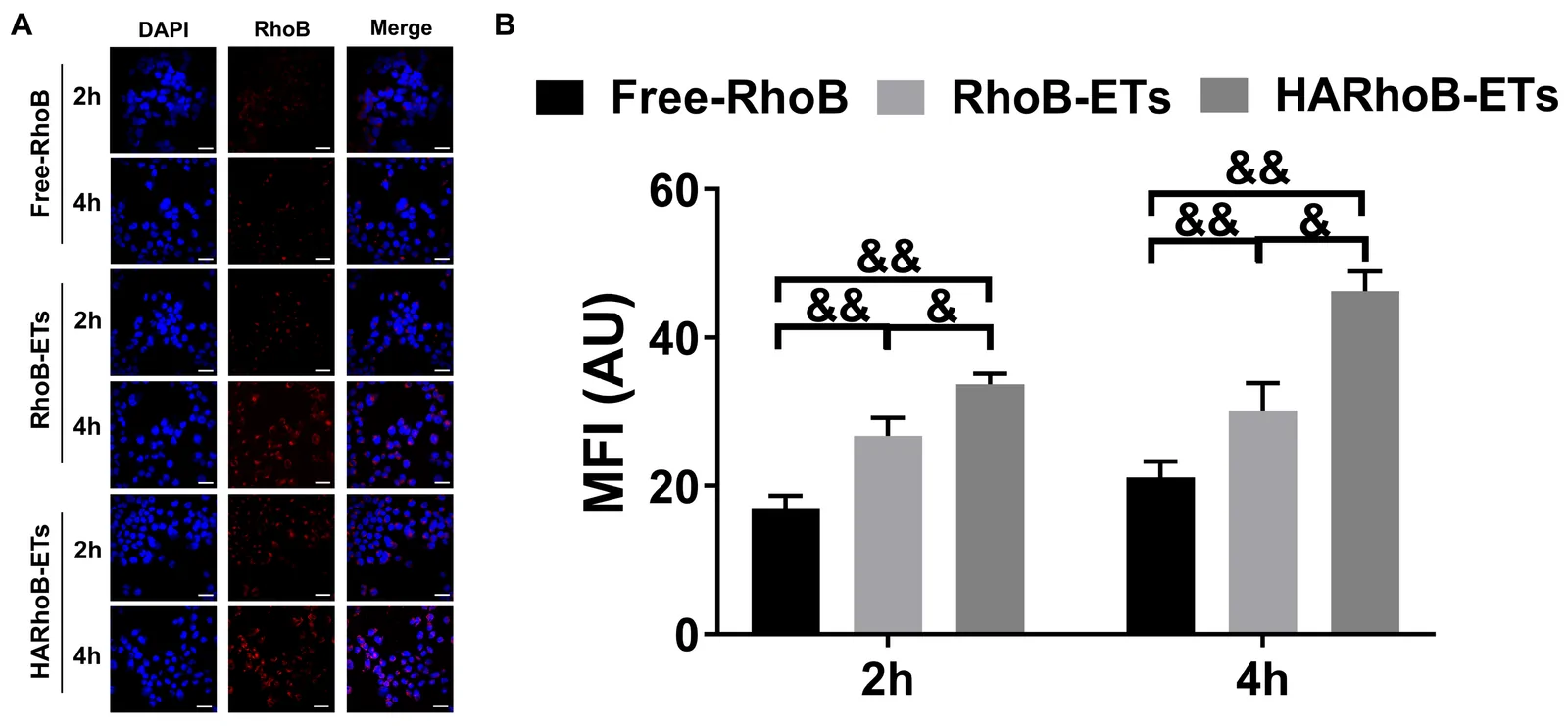
Cellular uptake of free RhoB, RhoB-ETs, and HARhoB-ETs in HaCaT cells. (**A**) Representative confocal fluorescence images obtained after incubation for 2 h and 4 h. (**B**) Flow cytometry analysis of cellular uptake after incubation for 2 h and 4 h. ^&^ *p* < 0.05, ^&&^ *p* < 0.01, Mean ± SD (n = 3). Scale bar = 50 μm.

Efficient intracellular delivery to keratinocytes is a key determinant of the local pharmacological activity of GA, since keratinocytes are major effector cells involved in epidermal inflammation, barrier regulation, and sensory signaling. As shown in Figure 4, intracellular fluorescence increased over time in all groups, indicating progressive uptake from 2 to 4 h. Both ethosomal formulations showed markedly stronger fluorescence than free RhoB at each time point, and HARhoB-ETs consistently exhibited the highest signal. These findings suggest that the improved cutaneous localization observed in the skin retention study was accompanied by enhanced cellular internalization of the nanocarrier system, suggesting that HA surface engineering improved the interaction and internalization of the ethosomal delivery system at both tissue and cellular levels. The enhanced internalization of RhoB-ETs relative to free RhoB is likely related to the nanoscale vesicular structure and the cationic surface, both of which may promote close contact with cell membranes and facilitate endocytic processes. The further increase observed with HARhoB-ETs indicates that HA did not merely improve formulation biocompatibility, but also contributed functionally to keratinocyte interaction.

**Figure 5:**
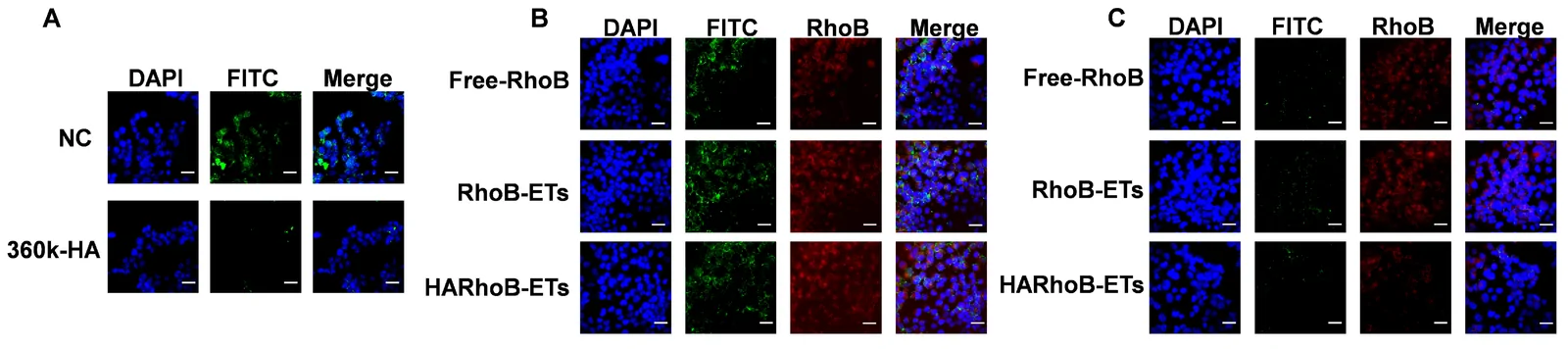
Effect of HA pre-saturation on the cellular uptake of HA-modified ethosomes in HaCaT cells. (**A**) Representative confocal images showing the uptake of FITC-HA in cells with or without preincubation with excess free HA. Representative confocal images showing the uptake of free RhoB, RhoB-ETs, and HARhoB-ETs in the absence (**B**) or presence (**C**) of HA pre-saturation. Data are presented as mean ± SD (n = 3). Scale bar: 50 μm.

To further examine the basis of this enhancement, competitive HA pre-saturation experiments were performed. Pretreatment with excess free HA markedly reduced the uptake of FITC-HA by HaCaT cells (Figure 5A), suggesting competitive occupation of HA-binding sites on the cell surface. Consistent with this observation, HA pre-saturation substantially attenuated the intracellular fluorescence of HARhoB-ETs, whereas only minimal changes were observed for free RhoB and unmodified ethosomes (Figure 5B,C and Figure S2). CD44 is a major HA receptor expressed on keratinocytes and has been widely implicated in HA-mediated cellular adhesion and internalization [46]. Therefore, the pronounced reduction in HARhoB-ET uptake following HA pre-saturation supports the involvement of HA receptor-mediated recognition in the enhanced cellular interaction of HAGA-ETs. However, because HA can interact with multiple HA-binding proteins and the present competitive assay does not directly interrogate CD44 function, the contribution of CD44 to this HA-dependent cellular interaction remains to be determined. Interestingly, this competitive inhibition pattern closely mirrors our previous findings obtained with another HA-functionalized cationic nanocarrier [32], in which free HA pre-saturation similarly reduced keratinocyte uptake. The reproducibility of this phenomenon across two distinct carrier systems suggests that the uptake-promoting effect of surface HA is unlikely to be formulation-specific, but instead represents a more general characteristic of HA-mediated surface engineering. Improved cellular interaction provides a plausible explanation for the enhanced pharmacological activity observed in the subsequent experiments. Increased intracellular delivery is expected to facilitate more efficient exposure of keratinocytes to GA, thereby contributing to the superior suppression of inflammatory mediators, restoration of barrier-related proteins, and regulation of neurosensory-associated pathways observed for HAGA-ETs. Further studies using CD44-specific blocking antibodies or gene-silencing approaches are warranted to clarify the receptor-level mechanism.

### 3.6. Cellular Protective and Anti-Inflammatory Activity

**Figure 6:**
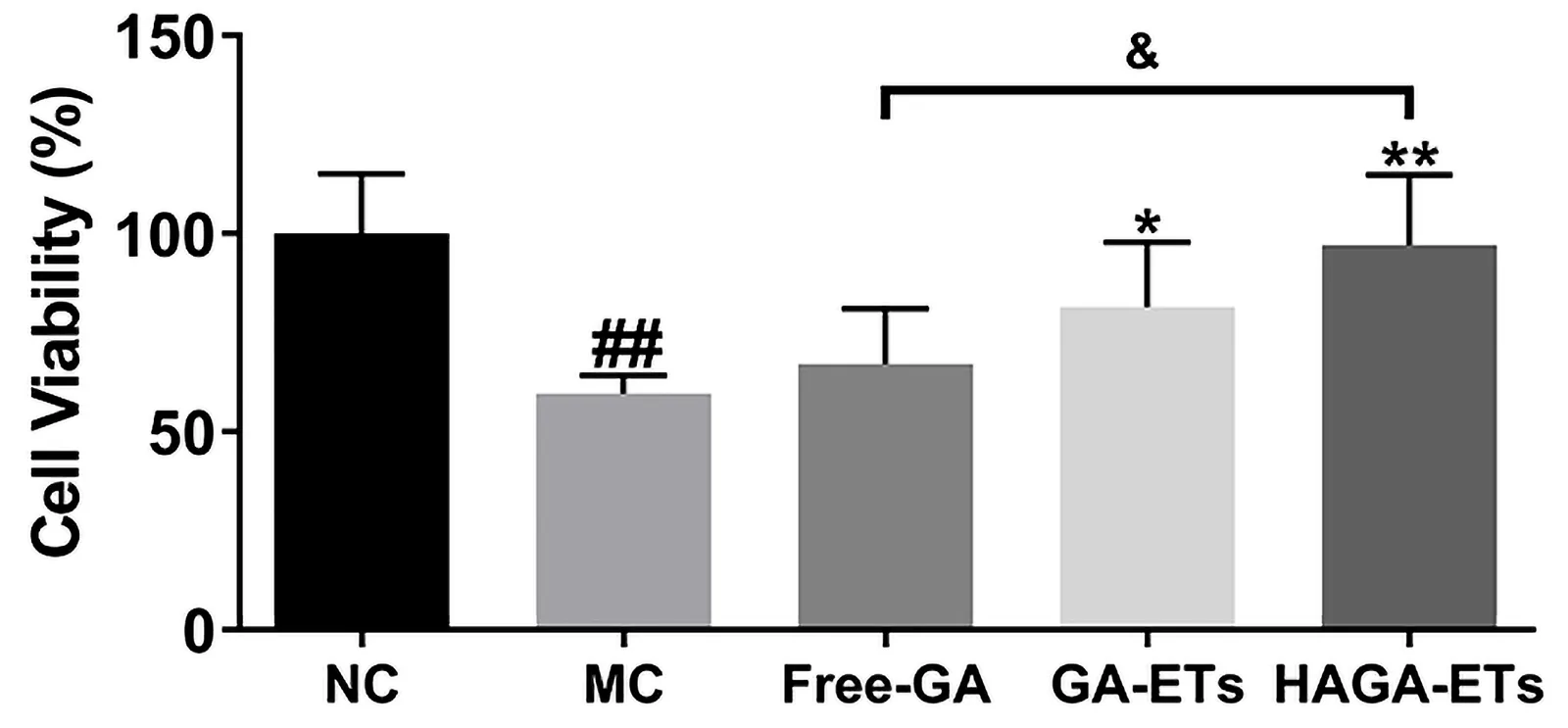
Protective effects of different formulations on the viability of TNF-α/IFN-γ-stimulated HaCaT cells. ^##^ *p* < 0.01 vs. NC, * *p* < 0.05 and ** *p* < 0.01 vs. MC, ^&^ *p* < 0.05.

A TNF-α/IFN-γ stimulated HaCaT model was established to evaluate the protective and anti-inflammatory effects of the formulations under inflammatory conditions. As shown in Figure 6, stimulation significantly reduced cell viability compared with the normal control, confirming successful establishment of a cell injury/inflammation model. Free-GA showed only a slight, non-significant increase in viability, whereas GA-ETs and HAGA-ETs significantly improved cell viability under inflammatory stimulation, with HAGA-ETs showing the most pronounced protective effect. This enhanced protective effect is likely associated with improved GA availability mediated by ethosomal encapsulation and HA surface engineering. Free-GA possesses known anti-inflammatory activity, but its cellular efficacy is limited by poor solubility and insufficient intracellular delivery. Encapsulation in ethosomes improved performance, presumably by increasing cell-associated transport and local drug availability. HA modification further enhanced this effect, which is consistent with the superior uptake behavior observed earlier. Thus, the greater viability recovery induced by HAGA-ETs likely reflects a combination of improved drug delivery efficiency and better formulation tolerability.

**Figure 7:**
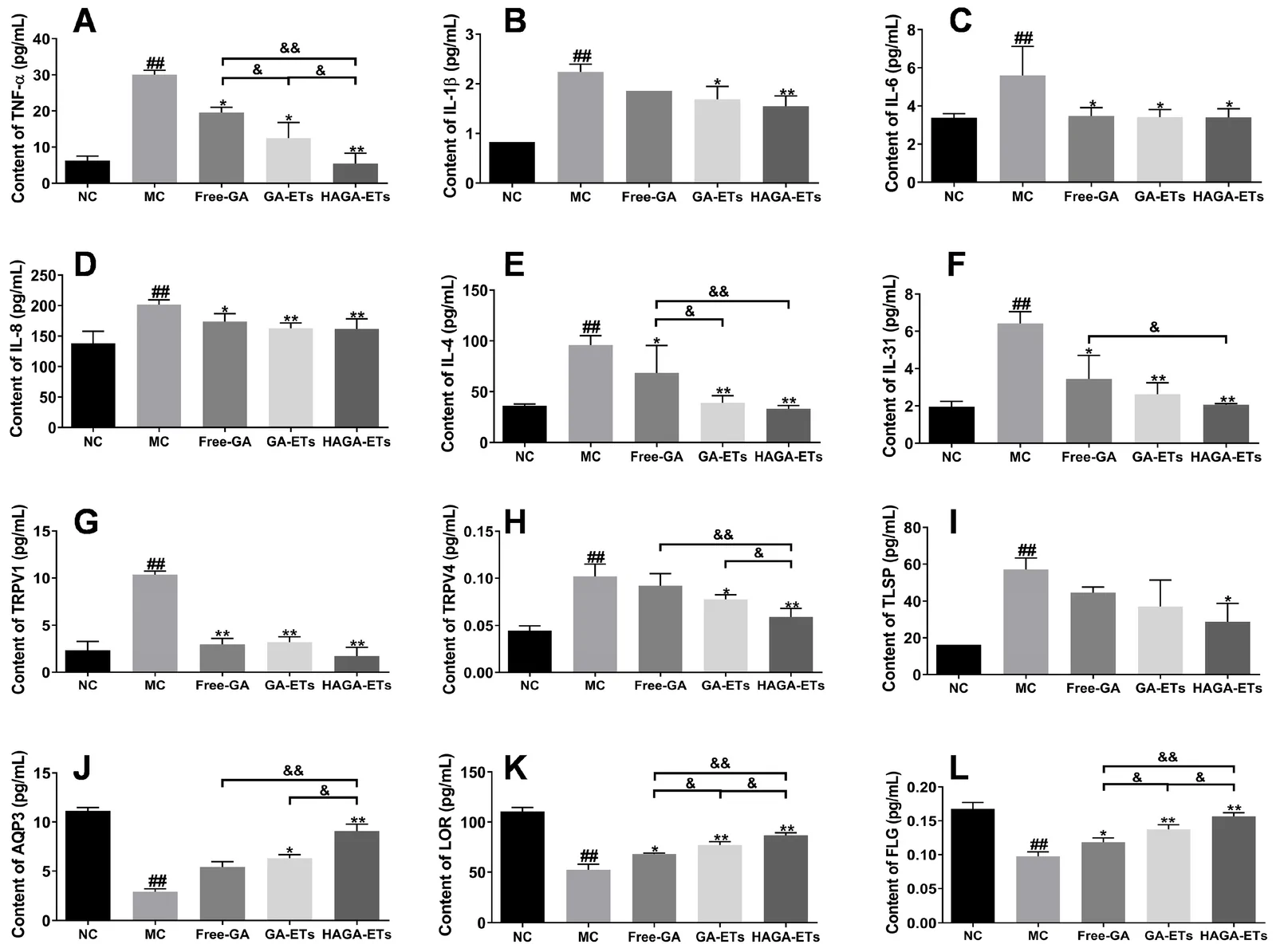
Effects of different formulations on inflammatory, barrier-related, and hyper-reactivity-associated markers in TNF-α/IFN-γ-stimulated HaCaT cells. Quantitative analysis of (**A**–**D**) pro-inflammatory cytokines (TNF-α, IL-1β, IL-6, and IL-8), (**E**–**I**) hypersensitivity-related mediators (IL-4, IL-31, TRPV1, TRPV4, and TSLP), and (**J**–**L**) barrier-associated proteins (AQP3, LOR, and FLG) after treatment with different formulations. ^##^ *p* < 0.01 vs. NC; * *p* < 0.05, ** *p* < 0.01 vs. MC; ^&^ *p* < 0.05, ^&&^ *p* < 0.01. Mean ± SD, n = 5.

To further evaluate anti-inflammatory activity, the levels of representative pro-inflammatory cytokines were measured by ELISA. As shown in Figure 7A–D, TNF-α/IFN-γ stimulation significantly increased the secretion of TNF-α, IL-1β, IL-6 and IL-8 in HaCaT cells. Treatment with GA-containing formulations attenuated these mediators to varying degrees, with HAGA-ETs showing the strongest overall inhibitory effect. Notably, although Free-GA reduced TNF-α, IL-6, and IL-8, its effect on IL-1β was not statistically significant; in contrast, both GA-ETs and HAGA-ETs significantly suppressed IL-1β secretion.

The broad suppression of these cytokines indicates that HAGA-ETs attenuated the inflammatory phenotype of stimulated keratinocytes rather than modulating a single inflammatory mediator. TNF-α and IL-1β are key upstream mediators that initiate and perpetuate local inflammatory signaling, whereas IL-6 and IL-8 are strongly associated with downstream inflammatory propagation and recruitment-related responses [47]. Simultaneous inhibition of these mediators therefore suggests a more comprehensive regulation of the inflammatory cascade. Importantly, these findings should be interpreted together with the preceding delivery studies. The superior anti-inflammatory activity of HAGA-ETs is unlikely to reflect an alteration in the intrinsic pharmacological properties of GA, but rather improved skin delivery and intracellular drug availability afforded by ethosomes and HA surface engineering. Enhanced cellular uptake is expected to increase the effective exposure of keratinocytes to GA, thereby translating its intrinsic anti-inflammatory activity into greater cellular efficacy. Together with the improved cell viability data, these results demonstrate that the enhanced biological performance of HAGA-ETs primarily arises from optimized drug delivery rather than changes in the pharmacological activity of the drug itself.

### 3.7. Regulation of Hyper-Reactivity and Allergic Sensitization-Related Markers

Sensitive skin is characterized not only by inflammation but also by abnormal sensory responsiveness and susceptibility to neuroimmune activation. To capture this aspect, IL-4, IL-31, TRPV1, TRPV4, and TSLP were measured in stimulated HaCaT cells. As shown in Figure 7E–I, all five markers were significantly elevated in the model group, indicating that the TNF-α/IFN-γ stimulation model reproduced features relevant to neuroimmune activation and sensory hyper-reactivity in addition to conventional inflammation. Treatment with Free-GA, GA-ETs, and HAGA-ETs reduced these mediators to different extents, with HAGA-ETs again showing the strongest effect. Notably, only HAGA-ETs significantly suppressed TSLP expression, whereas Free-GA and GA-ETs exhibited only downward trends without statistical significance.

This pattern highlights that the therapeutic activity of HAGA-ETs extends beyond conventional suppression of inflammatory cytokines. IL-4 is a representative Th2-associated cytokine linked to allergic-type immune skewing, and IL-31 is closely associated with pruritus and neuroimmune communication [18]. TSLP is a keratinocyte-derived alarmin that functions upstream in the initiation and amplification of allergic responses [48]. Meanwhile, TRPV1 and TRPV4 are key sensory ion channels involved in the perception of burning, stinging, and itching, and their abnormal activation is considered a hallmark of sensory hyper-reactivity in sensitive skin. The concurrent downregulation of these markers therefore suggests that HAGA-ETs regulate several pathological processes closely associated with the clinical manifestations of sensitive skin, rather than simply suppressing general inflammation. These findings are particularly relevant because the clinical burden of sensitive skin is largely driven by subjective symptoms, including burning, stinging, and itching, which cannot be fully explained by conventional inflammatory cytokines alone. By simultaneously attenuating inflammatory, neuroimmune, and sensory hyper-reactivity-associated markers, HAGA-ETs demonstrated a pharmacological profile that is more closely aligned with the current understanding of sensitive skin pathophysiology. Together with the anti-inflammatory findings presented before, these results further suggest that HA surface engineering enhances the local therapeutic efficacy of GA beyond what can be achieved by improving anti-inflammatory activity alone.

### 3.8. Effects on Barrier-Related Markers

Barrier dysfunction is another key feature of sensitive skin. To determine whether the formulations could improve barrier-associated functions in keratinocytes, the expression levels of AQP3, LOR, and FLG were measured. As shown in Figure 7J–L, TNF-α/IFN-γ stimulation significantly reduced the levels of AQP3, LOR, and FLG, indicating disruption of barrier-related homeostasis.

Treatment with Free-GA, GA-ETs, and HAGA-ETs increased the expression of these markers, with HAGA-ETs showing the most pronounced restorative effect. However, the increase in AQP3 in the Free-GA group was not statistically significant. AQP3 is essential for epidermal water transport and skin hydration, whereas LOR and FLG are major structural proteins required for cornified envelope formation and stratum corneum integrity [49]. The coordinated restoration of these three markers therefore suggests an overall improvement in epidermal barrier homeostasis rather than recovery of a single barrier-associated protein. The superior ability of HAGA-ETs to restore AQP3, LOR, and FLG is consistent with its enhanced intracellular delivery and indicates that improved local drug availability translated into more effective regulation of barrier-associated functions.

For sensitive skin, this finding has particular therapeutic relevance. Barrier damage is not only a consequence of inflammation but also a driver of further irritant penetration, inflammatory activation, and sensory aggravation. Accordingly, restoration of barrier integrity has the potential to interrupt this self-amplifying cycle rather than merely alleviate its downstream consequences. The superior recovery of AQP3, LOR, and FLG induced by HAGA-ETs therefore suggests that HA surface engineering enhanced the capacity of GA to restore epidermal barrier function through improved local delivery. Together with the anti-inflammatory and neuroimmune-regulating effects described in the preceding sections, these findings demonstrate that HAGA-ETs address several key pathological features of sensitive skin, including inflammation, barrier dysfunction, and sensory hyper-reactivity. Rather than representing independent pharmacological effects, these improvements collectively support the rationale that optimizing local drug delivery through HA surface engineering can enhance the overall therapeutic efficacy of GA for sensitive skin.

### 3.9. Western Blot Analysis

**Figure 8:**
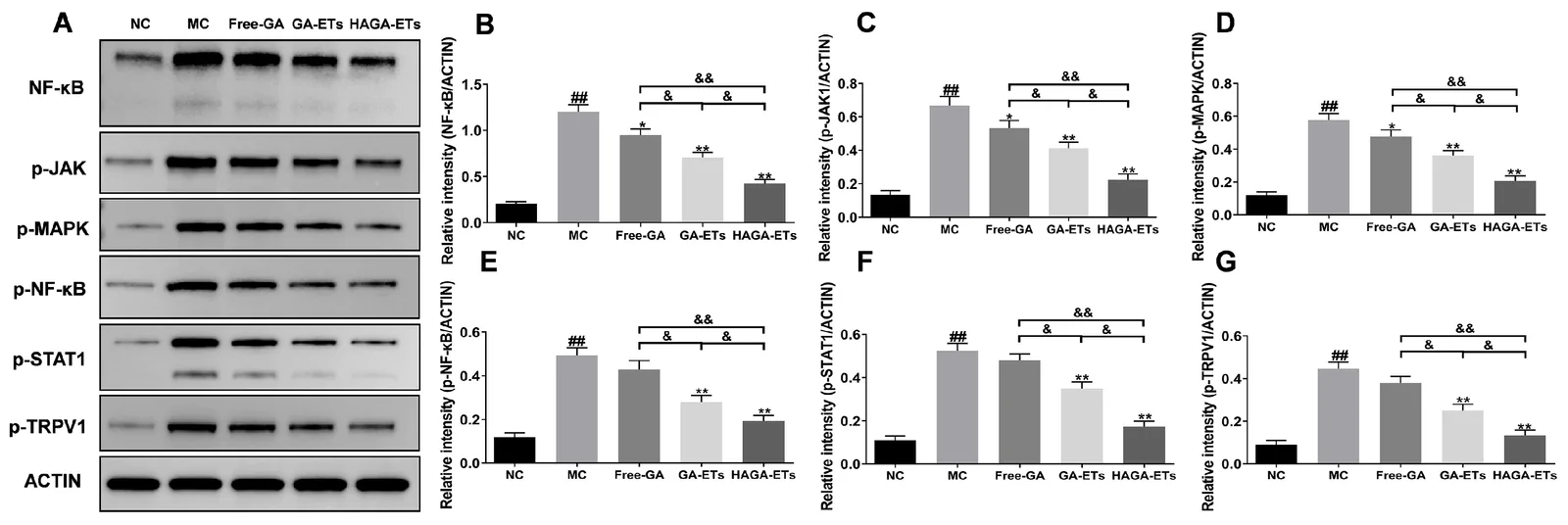
Effects of different formulations on inflammation- and hyper-reactivity-related signaling proteins in TNF-α/IFN-γ-stimulated HaCaT cells. (**A**) Representative Western blot bands. Quantitative analysis of (**B**) NF-κB, (**C**) p-JAK1, (**D**) p-MAPK, (**E**) p-NF-κB, (**F**) p-STAT1, and (**G**) p-TRPV1 relative abundance. The expression level of p-NF-κB was normalized to total NF-κB, whereas p-JAK1, p-MAPK, p-STAT1, and p-TRPV1 were normalized to β-actin as the loading control. The latter measurements represent the relative abundance of the phosphorylated protein forms rather than phosphorylation ratios. ^##^ *p* < 0.01 vs. NC; * *p* < 0.05, ** *p* < 0.01 vs. MC; ^&^ *p* < 0.05, ^&&^ *p* < 0.01. Mean ± SD, n = 3.

To further investigate the molecular basis underlying the anti-inflammatory and anti-hyper-reactivity effects of the formulations, key signaling proteins were analyzed by Western blot. As shown in Figure 8B–G, the relative abundance of NF-κB and the phosphorylated forms of JAK1, MAPK, NF-κB, STAT1, and TRPV1 was markedly increased in the model group compared with the normal control, suggesting enhanced involvement of multiple inflammation- and sensitivity-related signaling pathways in HaCaT cells following TNF-α/IFN-γ stimulation. Treatment with GA-ETs partially reduced the relative abundance of these phosphorylated proteins to different extents, with HAGA-ETs consistently showing the greatest reduction across the measured targets.

MAPK and NF-κB are central pathways involved in the transcriptional regulation of pro-inflammatory mediators, including TNF-α, IL-6, and IL-1β, and their overactivation is closely associated with cutaneous inflammatory responses [50]. The JAK1/STAT1 pathway contributes to cytokine-driven inflammatory signaling in keratinocytes, whereas TRPV1 is a key sensory receptor implicated in burning, stinging, and itch-associated responses [51]. The concurrent changes in these signaling-related proteins were consistent with the reduced inflammatory cytokine production, restoration of barrier-related markers, and attenuation of hyper-reactivity-associated mediators observed following HAGA-ET treatment, providing further mechanistic support for its enhanced biological activity. These findings suggest that HAGA-ETs may exert their enhanced biological effects through coordinated modulation of multiple interconnected signaling pathways rather than through a single molecular target. Such coordinated regulation is consistent with the multifactorial nature of sensitive skin, in which inflammatory activation, barrier dysfunction, and sensory hyper-reactivity are closely interconnected. It should be noted that the phosphorylated forms of JAK1, MAPK, STAT1, and TRPV1 were evaluated relative to β-actin, whereas their corresponding total protein levels were not examined. Thus, the observed changes represent alterations in the relative abundance of the phosphorylated protein forms. Future studies incorporating parallel analysis of the corresponding total proteins and phosphorylation status will further clarify the molecular mechanisms underlying the enhanced efficacy of HAGA-ETs.

### 3.10. Anti-Inflammatory Effect in the 3D Skin Model

**Figure 9:**
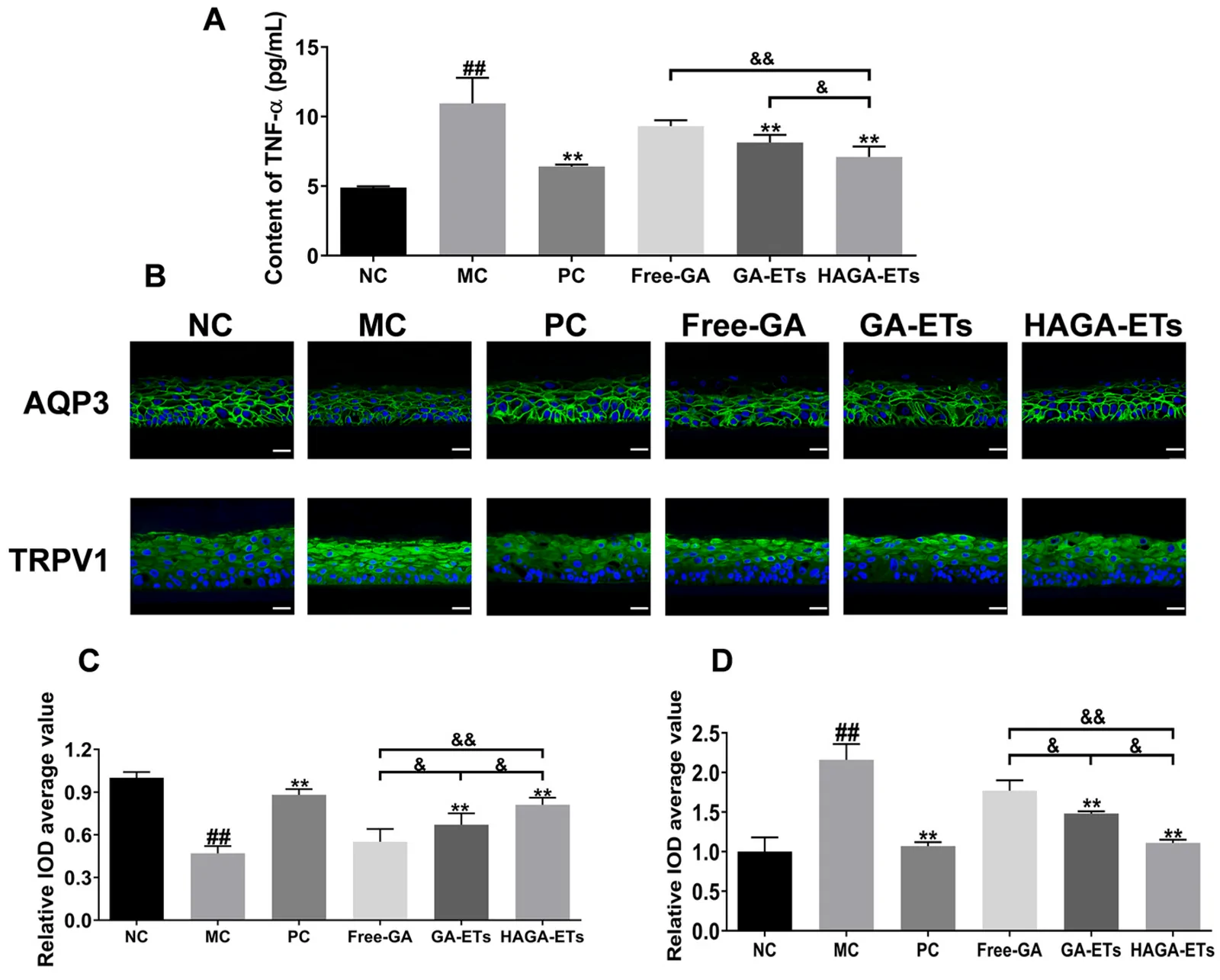
Validation of anti-inflammatory, barrier and hyper-reactivity-regulating effects in the 3D skin model. (**A**) TNF-α levels in tissue supernatants measured by ELISA. (**B**) Representative immunofluorescence images of AQP3 and TRPV1 in 3D epidermal tissues. Quantitative analysis of (**C**) AQP3 and (**D**) TRPV1 expression in 3D skin models following SLS stimulation. WY14643 was used as the positive control for AQP3, and trans-4-tert-butylcyclohexanol was used as the positive control for TRPV1. Nuclei were counterstained with DAPI (blue). ^##^ *p* < 0.01 vs. NC, ** *p* < 0.01 vs. MC, ^&^ *p* < 0.05, ^&&^ *p* < 0.01. Scale bar: 50 μm.

Compared with monolayer keratinocyte cultures, 3D skin models more closely recapitulate the structural and functional characteristics of the epidermal microenvironment. To further determine whether the advantages observed in HaCaT cells could be translated to a tissue-level model, the biological activity of the formulations was evaluated using an SLS-induced 3D skin model. The anti-inflammatory activity of the formulations was first evaluated by measuring TNF-α levels in the culture supernatant. As shown in Figure 9A, the TNF-α level in the model group was markedly higher than that in the normal control, confirming successful induction of inflammation in the 3D skin model. Treatment with Free-GA, GA-ETs, and HAGA-ETs reduced TNF-α secretion to different extents, with inhibition rates of 27.03%, 47.03%, and 63.16%, respectively. Among all formulations, HAGA-ETs exhibited the greatest anti-inflammatory efficacy. The improved anti-inflammatory activity observed in the 3D model is consistent with the cytokine findings in HaCaT cells, indicating that the enhanced intracellular delivery achieved by HA surface engineering remained effective in a structurally more complex epidermal model. These results suggest that the delivery advantage of HAGA-ETs is preserved beyond monolayer cell cultures and can be translated into improved tissue-level pharmacological activity.

### 3.11. Effect on Barrier-Related Markers in the 3D Skin Model

AQP3 immunofluorescence staining was performed to assess barrier-related effects. As shown in Figure 9B, AQP3 expression in the model group was significantly lower than that in the normal control, indicating that SLS treatment impaired epidermal barrier-associated homeostasis. Treatment with Free-GA, GA-ETs, and HAGA-ETs improved AQP3 expression, with HAGA-ETs showing the most pronounced restorative effect. Quantitative analysis showed barrier recovery rates of 15.09%, 37.74%, and 64.15% for Free-GA, GA-ETs, and HAGA-ETs, respectively. These findings corroborate the barrier-restorative effects observed in HaCaT cells and further demonstrate that improved intracellular delivery of GA can be translated into recovery of tissue-level barrier-associated function. Since impaired epidermal hydration and barrier integrity are key pathological features of sensitive skin, restoration of AQP3 in the 3D model provides additional evidence supporting the potential of HAGA-ETs to improve barrier homeostasis under conditions that more closely resemble human skin.

### 3.12. Modulation of Hyper-Reactivity in the 3D Skin Model

TRPV1 immunofluorescence staining was used to evaluate the effect of the formulations on skin hyper-reactivity in the 3D model. As shown in Figure 9B, TRPV1 fluorescence intensity was markedly elevated in the model group compared with the normal control, indicating enhanced sensory-related reactivity after SLS stimulation. Treatment with the different formulations reduced TRPV1 expression to varying extents, with HAGA-ETs showing the strongest inhibitory effect. Quantitative analysis showed inhibition rates of 33.62%, 58.62%, and 90.52% for Free-GA, GA-ETs, and HAGA-ETs, respectively. TRPV1 is a key mediator of burning, stinging, and itch-associated sensory responses and has been implicated in the pathogenesis of sensitive skin. The marked suppression of TRPV1 in the HAGA-ET group therefore suggests that the formulation may alleviate not only inflammatory responses but also sensory-related hyper-reactivity. Importantly, this observation is in good agreement with the reduced expression of TRPV1, TRPV4, IL-31, and TSLP observed in HaCaT cells, indicating that the regulatory effects of HAGA-ETs on hyper-reactivity-associated pathways were reproducible at both the cellular and tissue levels.

Taken together, the present study demonstrates that HA surface engineering represents an effective strategy for optimizing topical ethosomal delivery of GA. By improving local retention, keratinocyte interaction, and intracellular drug availability, HAGA-ETs achieved enhanced anti-inflammatory, barrier-restorative, and hyper-reactivity-regulating effects in both cellular and reconstructed skin models. Although the present study provides comprehensive evidence in cellular and reconstructed skin models, further evaluation in appropriate in vivo models will be important to confirm the long-term therapeutic efficacy, safety, and clinical translational potential of HAGA-ETs for sensitive skin management. Future studies could further explore the potential of this delivery platform for the topical delivery of other bioactive ingredients in broader dermatological applications, such as skin aging and pigmentation.

## 4. Conclusions

In conclusion, hyaluronic acid-coated glycyrrhetinic acid-loaded ethosomes (HAGA-ETs) were successfully developed through an electrostatic surface-engineering strategy as a topical delivery system for sensitive skin. The optimized formulation exhibited favorable physicochemical properties, sustained release behavior, enhanced skin retention, and improved keratinocyte uptake. More importantly, HA surface engineering improved local delivery performance and translated into superior biological activity, enabling HAGA-ETs to more effectively suppress inflammatory responses, regulate hyper-reactivity- and allergy-related mediators, and restore barrier-associated markers in both HaCaT cells and a reconstructed 3D skin model. Mechanistically, these effects were associated with attenuation of MAPK/NF-κB, JAK1/STAT1, and TRPV1-related signaling pathways. Collectively, the findings demonstrate that HA surface engineering represents an effective strategy for optimizing skin delivery of GA and highlight the potential of HAGA-ETs as a promising nanoplatform for the local management of sensitive skin.

## Figures and Tables

**Table 1:** Particle size, PDI, and zeta potential of GA-ETs and HAGA-ETs.

Sample	Size/nm	PDI	Zeta Potential (mV)	EE (%)	DL (%)
GA-ETs	83.04 ± 8.41	0.107 ± 0.006	+27.16 ± 0.72	93.4 ± 1.4	0.93 ± 0.12
HAGA-ETs	140.1 ± 7.67	0.115 ± 0.005	−20.25 ± 0.58	95.7 ± 1.3	0.96 ± 0.16

**Table 2:** Release equations of different formulations.

Group	Release Equation (*Q*(*t*))	R^2^
Free-GA	$Q\left(t\right)=93.78\times [1-exp(-({\frac{t}{8.15})}^{1.28})]$	0.999
GA-ETs	$Q\left(t\right)=58.46\times [1-exp(-({\frac{t}{12.62})}^{1.66})]$	0.998
HAGA-ETs	$Q\left(t\right)=43.51\times [1-exp(-({\frac{t}{15.18})}^{1.44})]$	0.999

Note: *Q*(*t*) is the cumulative release percentage (%), and *t* is the time in hours (h).

## Data Availability

The original contributions presented in the study are included in the article/Supplementary Materials; further inquiries can be directed to the corresponding authors.

## Supplementary Materials

The following supporting information can be downloaded at: https://www.mdpi.com/article/10.3390/pharmaceutics18091114/s1, Figure S1: Particle size distribution of GA-ETs and HAGA-ETs. DLS profile of GA-ETs (A) and HAGA-ETs (B). Figure S2: Effect of HA pre-saturation on the cellular uptake of HA-modified ethosomes in HaCaT cells. Intracellular fluorescence quantification in cells treated with free RhoB, RhoB-ETs, and HARhoB-ETs in the absence (A) or presence (B) of HA pre-saturation. & *p* < 0.05, && *p* < 0.01. Table S1: Physicochemical stability of GA-ETs and HAGA-ETs before and after 60 days of storage at 4 °C and 25 °C. Table S2: R^2^ values of different kinetic models.
