# Supplementary material for: A Hyaluronic Acid-Coated Ethosomal Delivery System for Improving the Topical Delivery of Glycyrrhetinic Acid in Sensitive Skin
Source: Pharmaceutics. 2026 Sep 4;18(9):1114. doi: 10.3390/pharmaceutics18091114 (PMC13610640; doi:10.3390/pharmaceutics18091114)
Supplement: Supplementary file 1 [file pharmaceutics-18-01114-s001.zip › pharmaceutics-4491293-supplementary.pdf]

Supplementary Materials

A Hyaluronic Acid-Coated Ethosomal Delivery System for Improving the Topical Delivery of Glycyrrhetinic Acid in Sensitive Skin

Yuling Wang[1,2], Shujing Ren[2], Jun Deng[3], Dan Luo[3], Rui Liu[3], Yu Zhou [1], Siyuan Chen[4], Wei Liu[1,3]

[1]College of Life Science and Technology, Huazhong University of Science and Technology, Wuhan, 430074, China;

[2]Research and Development Center, Bloomage Biotechnology Corporation Limited, Jinan, 250101, China;

[3]National Engineering Research Center for Nanomedicine, Huazhong University of Science and Technology, Wuhan, 430075, China;

[4]Research Institute for Biomaterials, Tech Institute for Advanced Materials Bioinspired Biomedical Materials & Devices Center, College of Materials Science and Engineering, Jiangsu Collaborative Innovation Center for Advanced Inorganic Function Composites, Suqian Advanced Materials Industry Technology Innovation Center, Nanjing Tech University, Nanjing, 211816, China

Correspondence: Wei Liu, Siyuan Chen
Email: wliu@hust.edu.cn; siyuan.chen@njtech.edu.cn

Table S1. Physicochemical stability of GA-ETs and HAGA-ETs before and after 60 days of storage at 4°C and 25°C

		Samples	Z-Average size (nm)	PDI	Zeta potential (mV)	EE (%)
Initial		GA-ETs	83.04	0.107	+27.16	93.4 ± 1.4
		HAGA-ETs	140.1	0.115	-20.25	95.7 ± 1.3
30 d	4 °C	GA-ETs	87.32	0.106	+25.87	92.7 ± 1.4
		HAGA-ETs	147.5	0.147	-21.63	95.4 ± 1.3
	25 °C	GA-ETs	89.44	0.155	+27.23	92.1 ± 1.4
		HAGA-ETs	167.2	0.175	-19.72	94.8 ± 1.3
60 d	4 °C	GA-ETs	91.35	0.228	+24.29	90.7 ± 1.4
		HAGA-ETs	157.2	0.222	-19.75	91.9 ± 1.3
	25 °C	GA-ETs	99.41	0.216	+23.94	89.5 ± 1.4
		HAGA-ETs	177.6	0.246	-18.49	90.2 ± 1.3

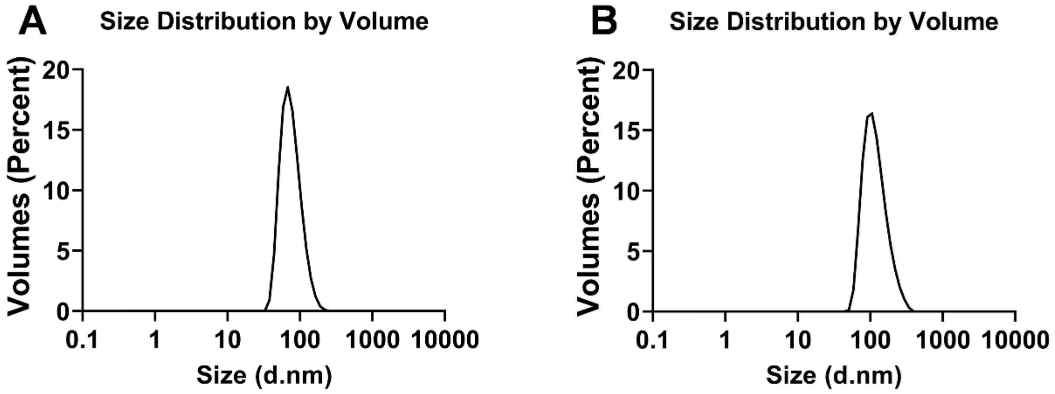

Figure S1. Particle size distribution of GA-ETs and HAGA-ETs. DLS profile of (A) GA-ETs and (B) HAGA-ETs.

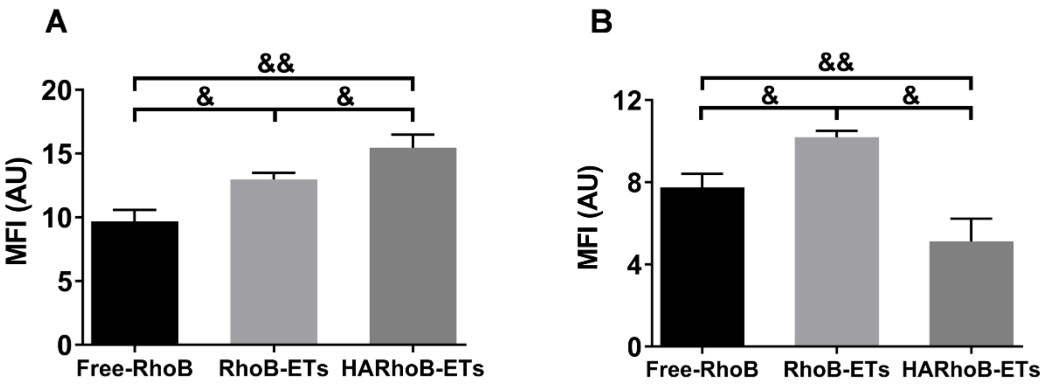

Figure S2. Effect of HA pre-saturation on the cellular uptake of HA-modified ethosomes in HaCaT cells. Intracellular fluorescence quantification in cells treated with free RhoB, RhoB-ETs, and HARhoB-ETs in the absence (A) or presence (B) of HA pre-saturation. $^{\&}p < 0.05$, $^{\&\&}p < 0.01$.

Table S2. R^2 values of different kinetic models

Group	Zero-order	First-order	Higuchi	Weibull
Free-GA	0.891	0.983	0.969	0.999
GA-ETs	0.898	0.970	0.989	0.998
HAGA-ETs	0.879	0.958	0.991	0.999
